# Novel Pharmaceutical Strategies for Enhancing Skin Penetration of Biomacromolecules

**DOI:** 10.3390/ph15070877

**Published:** 2022-07-16

**Authors:** Luyu Zhang, Zirong Dong, Wenjuan Liu, Xiying Wu, Haisheng He, Yi Lu, Wei Wu, Jianping Qi

**Affiliations:** Key Laboratory of Smart Drug Delivery of MOE, School of Pharmacy, Fudan University, Shanghai 201203, China; 20211030023@fudan.edu.cn (L.Z.); 19211030076@fudan.edu.cn (Z.D.); 21111030046@fudan.edu.cn (W.L.); wxyifd@163.com (X.W.); he_haisheng@fudan.edu.cn (H.H.); fd_luyi@fudan.edu.cn (Y.L.); wuwei@shmu.edu.cn (W.W.)

**Keywords:** skin delivery, macromolecules, ionic liquids, cell-penetrating peptides, nanoparticles, microneedles

## Abstract

Skin delivery of biomacromolecules holds great advantages in the systemic and local treatment of multiple diseases. However, the densely packed stratum corneum and the tight junctions between keratinocytes stand as formidable skin barriers against the penetration of most drug molecules. The large molecular weight, high hydrophilicity, and lability nature of biomacromolecules pose further challenges to their skin penetration. Recently, novel penetration enhancers, nano vesicles, and microneedles have emerged as efficient strategies to deliver biomacromolecules deep into the skin to exert their therapeutic action. This paper reviews the potential application and mechanisms of novel skin delivery strategies with emphasis on the pharmaceutical formulations.

## 1. Introduction

Transdermal delivery systems have undergone four decades of development since the first scopolamine transdermal patch was approved by the Food and Drug Administration (FDA) in the United States [1]. The skin delivery route constantly attracts academic and industrial attention for its unparallel advantages compared with oral delivery and hypodermic injection; these include the avoidance of drug degradation in the gastrointestinal tract and the hepatic first-pass effect; reduced fluctuation in plasma drug level; easy administration; good patient compliance; and immediate therapy termination via transdermal patch detachment in case of adverse side effects [2].

Meanwhile, macromolecular biologics, via the modalities of peptides, proteins, oligo-/poly-nucleotides, and polysaccharides, have taken up an increasing proportion of the available treatment over the years [3]. Biomacromolecules have high potential for transforming the current therapeutic regimen in treating skin diseases. For example, monoclonal antibodies of the immune checkpoints CTLA-4 (i.e., ipilimumab) and PD-1 (i.e., pembrolizumab and nivolumab) have been approved by the FDA for skin melanoma treatment as monotherapy, and trialed for combined therapy [4]. The gene silencing of tumor progression-related targets by siRNA may also serve a more durable purpose than the traditional small-molecule inhibitors. Other insightful research work has also shown the great potential of macromolecular biologics in treating chronic wounds [5,6,7], atopic dermatitis [8,9], psoriasis [10,11,12], etc.

Transcutaneous vaccination is rising, becoming a potent substitute for intramuscular inoculation to elicit a robust immune response with a lower antigen dose. The skin hosts abundant antigen-presenting cells in the dermis and viable epidermis, which, together with migratory T cells, contribute to the skin’s immunocompetence and can be referred to as skin-associated lymphoid tissue (SALT) [13]. Preliminary research has shown that transcutaneous vaccination is quite effective against infectious pathogens such as hepatitis B virus [14], *Streptococcus pneumoniae* [15], *Haemophilus influenzae* [16], and *Plasmodium falciparum* [17]. Additionally, antigen-encoded DNA, tumor-derived protein/peptide [18,19,20,21], and tumor cell lysate [22,23] have also been investigated as cutaneous vaccines for tumor immunotherapy, some of which have even entered clinical trials [24].

Nevertheless, poor skin permeability remains to be the primary downside of skin delivery. The compact structure of skin’s outermost layer, namely the stratum corneum (SC), sets rather stringent requirements for transdermal drug candidates. The famous Lipinski’s rule of five formerly proposed that poor drug-likeness for oral delivery is expected when the molecular weight (Mw) exceeds500, the number of hydrogen acceptors (NHAs) exceeds10, the number of hydrogen donors (NHDs) exceeds5, or the octanol–water partition coefficient (logP) exceeds5 [25]. This empirical rule can be extended to the skin delivery route [26]. Mw governs the partition coefficient, while logP reflects the SC–water partition. NHA and NHD indicate the interaction between drug molecules and the surfaces of corneocytes [27]. Accordingly, the physicochemical properties of biomacromolecules fail to meet the criteria for good drug-likeness in dermal or transdermal drug delivery. They are also extremely labile in their nature and prevalently necessitate parenteral delivery [28,29]. Therefore, an efficient penetration-enhancement strategy with low irritation and ease of use is the stepping stone to reach the full potential of dermal and transdermal biomacromolecule delivery. Currently, over 350 chemicals have been proposed as topical penetration enhancers, including surfactants, terpenes, sulfoxides, laurocapram, pyrrolidones, urea, fatty acids, etc. [30]. However, high potency often manifests with notable toxicity and skin irritation. Additionally, meddling with SC components could easily lead to skin dehydration and endogenous substance leakage [31]. Physical approaches using electrical, magnetic, photochemical, and ultrasonic waves have been investigated to enhance the skin penetration of therapeutics [32]. However, the cost and device portability of these physical approaches will eventually pose major hindrances during clinical uses. Therefore, physical approaches involving sophisticated devices will be intentionally left out in this review.

This review summarizes the recent development in enhancing the skin delivery of biomacromolecules, with highlights on rational formulation design and potential application. Novel penetration enhancers such as ionic liquids and cell-penetrating peptides as well as nano drug carriers will be elaborated. Even though a microneedle platform is more frequently assigned as a medical device or drug–device combination product, it is also included in this review to discuss the significance of microneedle formulation on drug-delivery behavior.

## 2. Physiological Barriers Hindering Skin Penetration of Biomacromolecules

**Figure 1:**
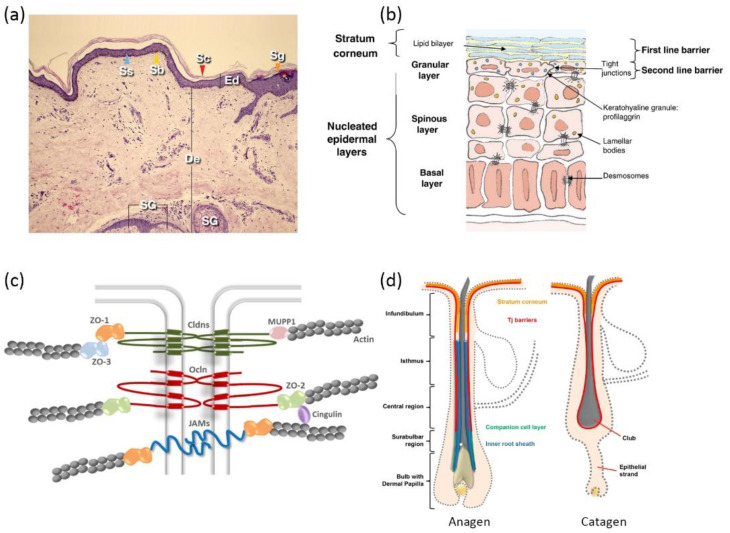
Schematic illustrations of skin: (**a**) histological cross-section of the skin (Ed—epidermis; Sc—stratum corneum; De—dermis; Sg—stratum granulosum; Ss—stratum spinosum; Sb—stratum basale; SG—sebaceous gland) (reproduced with permission from Arda et al., Clinics in Dermatology, adapted with permission from Ref.[49]. Copyright 2014 Elsevier); (**b**) epidermis structure (reproduced with permission from Baroni et al., Clinics in Dermatology; adapted with permission from Ref.[34]. Copyright 2012 Elsevier); (**c**) protein complexes of tight junctions (reproduced with permission from Basler et al., Journal of Controlled Release; adapted with permission from Ref.[46]. Copyright 2016 Elsevier); and (**d**) hair follicle structure of anagen phase and catagen phase (Reproduced with permission from Gorzelanny et al., Pharmaceutics; adapted with permission from Ref.[36]. Copyright 2020 MDPI).

The complex architecture of mammalian skin gives rise to its multi-faced functions, involving thermoregulation, sensation, metabolism, and immunization [33]. Most importantly, skin provides a bidirectional barrier against internal water or electrolyte loss as well as foreign insults [34]. Skin is generally comprised of three layers: the epidermis, dermis, and hypodermis (Figure 1a). The epidermis is outwardly composed of the *stratum basale* (SB), *stratum spinosum* (SS), *stratum granulosum* (SG), and *stratum corneum* (SC) [35], distinguished by the differentiation stages of keratinocytes (Figure 1b). Due to the existence of SC and tight junctions (TJs) [36,37,38], the epidermis has frequently been addressed as the rate-limiting mechanical barrier against the penetration of most drug molecules.

The SC is composed of 10–20 layers of non-viable cornified keratinocytes, which are more often termed as corneocytes [39]. Each corneocyte is encased in a hydrophobic cornified envelope that is tightly bound to the extracellular lipids, hindering the transdermal fluxes of hydrophilic substances [36,40]. SC lipids contain quasi-equimolar amounts of ceramides, free fatty acids, and cholesterol [41]. Ceramides found in the SC of human skin are considerably less polar than typical cell membrane lipids [42]. Generally, lamellar lipid layers in the SC exist in a densely packed orthorhombic phase (OR). The lipid chains in this phase adopt all-trans conformation and are packed in a rectangular crystalline lattice with no rotational or translational mobility [43]. Corneodesmosomes, the modified desmosomes in the SC, further contribute to the reinforcement of corneocyte cohesion [44]. Together, the skin lipid lamellae, cornified envelopes, and corneocytes constitute the “brick and mortar” structure, which is the skin’s first barrier against foreign insults as well as drug influxes [39].

TJs are found at the cell–cell borders in SG layers, which form a second barrier against the skin penetration of drug molecules by sealing the paracellular pathway. It is reported that TJ formation reduces the permeability of fluorescent tracer (332 Da) and dextran (40kDa) in a size-dependent manner [45]. TJs are dynamic protein complexes containing transmembrane proteins (e.g., claudins, occludins, and junctional adhesion molecules) as well as TJ plaque proteins (e.g., zonula occludens, and cingulin) [46] (Figure 1c). Claudins have been reported to be obligatory for the assembly of the TJ complex [47]. Mice with claudin-1 deficiency led to TJ barrier leakage to a ~600 Da tracer molecule [48].

Given the compact structure of epidermis, there exists three possible pathways for skin penetration, which can be designated as the paracellular pathway, transcellular pathway, and transappendageal pathway. The paracellular pathway is accomplished by diffusion into paracellular skin lipids, which is primarily favored by lipophilic small drug molecules. The transcellular pathway sets rigorous requirements for the physiochemical properties of drug molecules, since it involves consecutive partition and diffusion between hydrophilic and lipophilic domains [31]. Skin appendages take a rather limited role in the percutaneous penetration of most drug molecules, considering that they only take up a small proportion of the total skin area (<1%). However, various reports suggest that skin appendages (hair follicles in particular) appear to be an efficient penetration pathway and reservoir for macromolecules [50,51,52]. Skin penetration via hair follicles (HFs) could seemingly bypass skin barriers such as the SC. However, a detailed investigation of HF anatomy unveiled that TJs are still present from the upper infundibulum down to the central region for HFs in anagen (i.e., the growing phase), and TJs completely cover the club hair in the catagen and telogen phases (i.e., the regression and resting phases) (Figure 1d). Additionally, SC is also found in the infundibulum, forming a double barrier in this region [53]. The dermal glands generally form TJ barriers, as well, but uptake via glands is not preferred due to their nature of inside-out secretion [54].

## 3. Novel Pharmaceutical Strategies for Skin Penetration

Efficient skin penetration is a prerequisite for biomacromolecules to exert their localized or systemic pharmacological actions. Considering biocompatibility, ease of use and the potential for massive production, this review summarizes novel penetration-enhancing strategies for biomacromolecules. We highlight the impact of pharmaceutical formulation design on skin-penetrating behavior. Ionic liquids and Cell penetrating peptides are proposed as novel penetration enhancers to deliver biologics deep into the skin via the disturbance of the SC layer and cell membrane [55,56]. Nanosystems of various materials and structures also pose as versatile vesicles to encapsulate biomacromolecules for enhancing penetration or increasing skin accumulation [57]. Microneedles could bypass the SC barrier and achieve instant or controlled drug release via fine-tuning of the matrix material [58]. A plethora of pilot investigations are incorporated in this review to discuss the drug-delivery performance and the potential application in disease treatment.

### 3.1. Ionic Liquids (ILs)

ILs are commonly defined as molten salts with melting points under 100 °C. Empirically, ILs are composed of bulky, asymmetric organic cations and weakly coordinating anions, hindering strong ionic interaction and disturbing the crystal lattice to retain liquid form at room temperature [59]. Principle synthesizing methods are feasible for scale-up production, including direct acid–base neutralization, salt metathesis in proper solution, and solvent-free metathesis via melting or grinding [60]. When ILs are not synthesized strictly according to stoichiometric ratio, excess neutral substances are also present in the systems. The definition of such systems sometimes overlaps with deep eutectic solvents (DESs). A DES is a mixture that exhibits lower melting points than any of its components. DESs and ILs are favored by different researchers with trivial differences: “ILs” emphasize ionic interaction while “DESs” highlight the profound hydrogen bonding [61]. For convenience, this review will uniformly address such systems as ILs.

**Figure 2:**
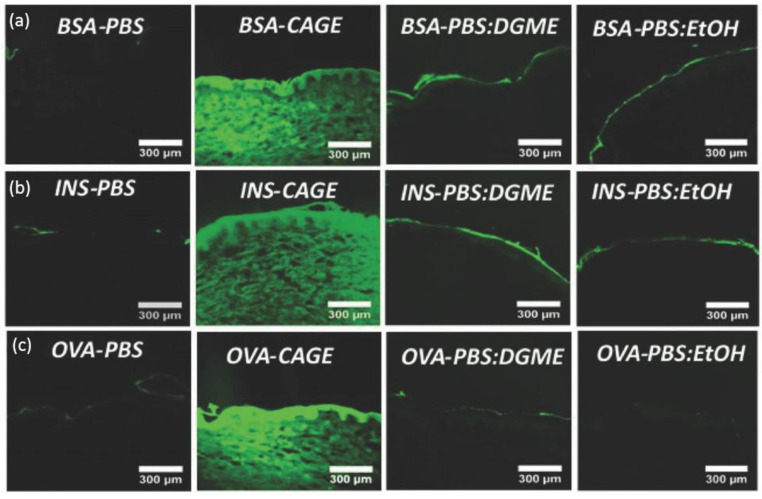
In vitro skin penetration of (**a**) FITC-BSA, (**b**) FITC-Insulin, and (**c**) FITC-OVA in PBS, CAGE, 50:50 (*v*/*v*) PBS/DGME, and 50:50 (*v*/*v*) PBS/ethanol, respectively. BSA—bovine serum albumin; INS—insulin; OVA—ovalbumin; PBS—phosphate buffered saline; DGME—diethylene glycol monoethyl ether (reproduced with permission from Banerjee et al., Advanced Healthcare Materials; adapted with permission from Ref.[55]. Copyright 2017 Wiley).

IL-incorporated drug-delivery systems come with the merits of enhanced solubility and permeability for both polar and non-polar drugs [62]. The solvation ability is largely credited to anions’ hydrogen bond forming and oxygen charge delocalization [60]. Erstwhile, research found that choline–geranate ILs (CAGE) could penetrate deep into the skin for deep-layer infection treatment with negligible toxicity to keratinocyte in mice [63]. Recent encouraging achievements were made by using ILs to enhance the skin penetration of biomacromolecules. Banerjee et al. firstly reported that CAGE could efficiently deliver bovine serum albumin (BSA, 66 kDa), ovalbumin (45 kDa), and insulin (5.8 kDa) deep into the epidermis and dermis of in vitro porcine skin in a time-dependent manner (Figure 2) [55]. However, the proportion of protein cargos in different skin layers may vary according to molecular weight. Compared to BSA, a larger portion of insulin was delivered into the epidermis, dermis, and receptor fluid after 24 h (80% for BSA and 93% for insulin). Protein-loaded ILs could further be integrated into biopolymeric film for improved mechanical strength [64]. CAGE could also facilitate the skin delivery of dextran with molecular weight up to 150k Da [65]. Choline-based ILs with other benign anions of different chain lengths and functional groups are also explored. Wu et al. reported that choline–malate IL could enhance the skin delivery of dextran two-fold [66], while choline–citric acid IL could enhance hyaluronic acid permeation five-fold [67]. Moreover, the phosphate groups of siRNA could be directly taken as anions to form novel IL entities with proper cationic moieties for the dual purposes of enhanced skin transport and cell transfection [68].

Given the amphiphilic nature, ILs based on choline–fatty acids were scrutinized as surface active reagents to incorporate hydrophilic biomacromolecules into a classic oil-based penetration enhancer such as isopropyl myristate (IPM) [69,70,71]. Tahara et al. recently reported that ILs derived from choline and oleic acid ([Ch][Ole]) facilitated the dispersion of antigenic peptides in IPM and ethanol (EtOH). The formulation showed little skin irritation, and the transdermal flux of peptide in IL/IPM/EtOH was 28- and 9-times higher than that in EtOH/PBS and EtOH/IPM, respectively [69]. The EtOH phase could be substituted with another hydrophilic IL derived from choline and propionic acid to robe the biologics before mixing with [Ch][Ole] and IPM [71]. A similar strategy was explored to disperse insulin in IPM for transdermal delivery, with [Ch][Ole] as a surfactant and Span-20 as a co-surfactant [70]. The IL-assisted microemulsion enabled higher bioavailability in systemic circulation and sustained insulin levels for a longer period (half-life > 24 h) compared to subcutaneous injection [70].

The latest work in this field pays extra attention to biomacromolecule stability during the formulation and drug delivering process. Tailor-made ILs with self-buffering capability could offer additional protection for macromolecules’ thermal, structural, and biological stability [72]. For example, the addition of a buffered choline–dihydrogen phosphate IL could substantially prolong the shelf-life of siRNA up to three months [73]. As another example, molecular simulation results indicated that CAGE could stabilize insulin by occupying the solvation shell of insulin, concomitantly exiling water molecules from the insulin surface [74]. Furthermore, an IL mixture of CAGE and choline–phenylpropanoic acid (CAPA) was designed to topically deliver siRNA for psoriasis treatment [10]. CAGE played the role of permeation enhancer, while CAPA retained the secondary structure of siRNA, probably owing to the intercalation between RNA base pairs and CAPA’s aromatic rings [10].

The mechanism behind ILs’ permeation enhancement is still in debate. Rudimentary theory involves SC lipid extraction by ILs [55,75]. ILs also enable SC lipids to transform from the orthorhombic phase to liquid crystalline packing, resulting in greater lipid fluidity [76]. In addition, an increase in unordered protein in SC was observed, while the deeper epidermis was not significantly affected [65]. Morphological examination found distinct gaps among lipid lamella, and the “brick and mortar” structure of SC became less densely packed after IL treatment [77]. The anion/cation ratio of ILs governs hydrogen bonding and ion pairing, which directly influences the viscosity, conductivity, and diffusivity of the IL formulation. Profound proton exchange also exists between the ions and the excess neutral components. Tanner et al. investigated the significance of ion ratio for CAGE’s penetration ability in the context of insulin delivery [75]. CAGE with an ion ratio of 1:1 exhibited the highest degree of ion pairing but the lowest permeation enhancement, presumably owing to the ions’ preference to interact with each other rather than with macromolecular drug or skin lipids. Moreover, the internal structure of ILs may alter when exposed to body fluid or water dilution. Taking CAGE as an example, a 25–50 vol% of water would transfer ILs into the lamellar phase, while further dilution would lead to micelle formation via aggregation of geranate anions’ hydrophobic chains [78]. However, current research works seldom discuss the influence of hydration state on the permeation enhancement of ILs.

### 3.2. Cell-Penetrating Peptides (CPPs)

CPPs comprise a family of short peptides (5–30 amino acids) that have demonstrated impressive penetration across bio-barriers without damaging the membrane integrity [79]. Commonly used CPPs bear positive charges owing to arginine and lysine residues (such as TAT, DPV3, and R8). Around 44% of CPPs are amphiphilic, characterized by a proline-enriched sequence or a combination of hydrophobic and polar regions in either the first or secondary structure (such as BPrPr, pVEC, and ARF). Hydrophobic CPPs (such as K-FGF, C105Y, gH625) only take up 15% of the whole CPP family [80].

A naturally derived protein bank contains rich resources for CPP design, such as heparin-binding proteins, signal peptides, nucleic acid-binding proteins, and anti-microbial peptides [81]. Certain CPPs could, additionally, serve as ligands for target delivery, bringing extra benefits for treating malignant skin diseases such as melanoma [82]. However, extracting CPPs from their original proteins may alter their secondary conformation and result in a loss of penetration ability. Artificially designed CPPs have further diversified the CPP bank by introducing novel sequences, unnatural amino acids [83] or even modified shapes such as dendrimers [84] and cyclic rings [85]. These exquisitely designed CPPs substantially improve the cargo-loading efficiency as well as resistance against cellular degradation. Additionally, carbohydrate scaffolds modified with guanidine could substitute the typical peptide backbone as transdermal enhancers. For example, a sorbitol-based carrier with 8 units of guanidine (Sor-G8) was shown to be more efficient than the arginine-rich CPP, R8, regarding skin-penetration ability [86].

The inchoate formulation strategy adopts a simple physical complexation of CPPs and biomacromolecules via electrostatic or hydrophobic interaction. An excessive CPP ratio is preferred to form positively charged nano-scale complexes. Chen et al. identified a cyclic CPP named TD-1(ACSSSPSKHCG) via phage display, which facilitated the transdermal delivery of insulin via simple coadministration and suppressed blood glucose levels for at least 11 h [87]. However, physical complexation via bulk mixing unavoidably results in poly-dispersed particle size or heterogeneous nanostructures, which potentially hinder transdermal efficacy. As for chemical conjugation, proteins and oligonucleotides are compatible for direct CPP conjugation via amino/sulfide bonds or PEG linkage. However, the multiple active sites on the cargos may result in uncontrollable conjugation.

Recombinant protein is a rather advanced strategy to achieve site-specific and homogeneous CPP conjugation [88]. For instance, TD-1 could be incorporated to construct a fusion protein with human epidermal growth factor (hEGF) without deactivating the latter. The resulting fusion protein TD-1-hEGF exhibited higher skin-penetration ability than the simple co-administration of TD-1 and hEGF [79]. In another case, Gautam et al. utilized a novel human-derived arginine-rich CPP, IMT-P8, to construct a fusion protein with green fluorescence protein (GFP) and proapoptotic peptide KLA. A large amount of IMT-P8-GFP and IMT-P8-KLA was detected in viable epidermis and hair follicles, and the delivery efficiency of IMT-P8 was even higher than the classical TAT [89]. Another approach is to construct a CPP fusion protein with a ligand to enhance target delivery within the skin. Considering that epidermal growth factor (EGF) is overexpressed in many tumor cells, a fusion protein of EGF (ligand) and SPACE (CPP) was constructed to deliver antineoplastic siRNA [90]. Moreover, it is a common strategy to attach CPPs to nanovesicles for enhanced skin delivery, which will be discussed in the next section.

**Figure 3:**
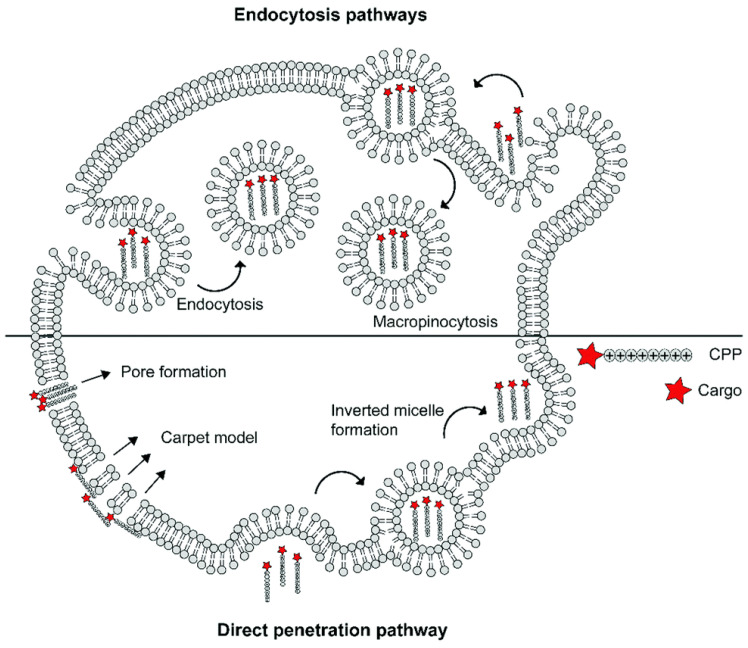
Schematic summary of different mechanisms underlying the cellular uptake of cell-penetrating peptide (reproduced with permission from de Jong et al., RSC Chemical Biology; published by RSC Publishing, 2020) [96].

The specific mechanisms of CPP–cargo translocation are contingent on several factors, including the nature of cargos (type, size, and charge), the physicochemical properties of CPPs (molecular weight, charge delocalization, and hydrophobicity), and even the experimental condition (pH, temperature, CPP concentration, and cell line types). Generally, cellular uptake is initiated by cellular contact between the cationic CPP and anionic phospholipid bilayers, glycosaminoglycans, or proteoglycans on the cell surface [56]. Thereafter, the internalization of CPP-cargo complexes can be divided into an energy-independent pathway (i.e., direct translocation) and an energy-dependent pathway (i.e., endocytosis) (Figure 3). Three models of energy-independent translocation were proposed: (1) the inverted micelle model [91]; (2) the carpet-like model [92]; and (3) the transient-pore model [93]. As for the active penetration pathway, endocytosis can be further divided into phagocytosis and pinocytosis (including macropinocytosis, clathrin-mediated endocytosis, caveolae-mediated endocytosis, and clathrin- and caveolae-independent endocytosis). The detailed mechanism of CPP-cargo internalization is beyond the scope of this review, but can be read about in the newly published literature [56,80,81,94,95].

### 3.3. Nanotechnology

Nanotechnology is trending in all aspects of drug delivery for increased efficacy and decreased toxicity, drawing attention to its unique nano-size effect as well as its versatility in surface modification. Having gone through several decades of development, nano-scale drug-delivery vesicles (<500 nm) now adopt different forms including nanoparticles, nanotubes, nanoemulsions, liposomes, micelles, etc. Endowed by their physio-chemical properties and surface modification, nanovesicles serve multiple purposes of enhanced drug solubility and permeability, target enrichment, bio-response to their microenvironment, and evasion from immune elimination. Nano-encapsulation also provides effective protection for liable biologic therapeutics against ambient degradation factors. Therefore, various nanovesicles have been reported to load biomacromolecules for enhanced skin penetration and increased skin accumulation (Table 1).

#### 3.3.1. Inorganic Nanoparticles

Inorganic nanoparticles in the forms of metallic nanoparticles, mesoporous silica nanoparticles, quantum dots, and carbon tubes have been proposed as transdermal vesicles. Gold nanoparticles (AuNPs) are rigid metallic nanoparticles that often come with a small size (<30 nm) and high compatibility. Surface modification can easily be achieved via Au-S interaction. AuNPs have been shown to disrupt phospholipid layers and modulate lipid-phase transitions, transiently increasing skin porosity and lipid fluidity [132]. Chen et al. attached vascular endothelial growth factor (VEGF) to AuNPs via a pegylated linker. Even with negative surface charge, such vesicles of approximately 20nm were able to deliver VEGF to the subcutaneous region and promoted subcutaneous angiogenesis 7 days after the topical treatment [102]. Huang et al. investigated the potential transcutaneous immunization effect via simple co-administration of ovalbumin and superfine AuNPs (<10 nm) [104]. The macromolecules were mainly detected in the epidermis with no biased accumulation in hair follicles, slowly migrating to the deeper layers. The topical treatment elicited robust and consistent immunization, with anti-OVA IgG levels comparable to intramuscular inoculation [104]. AuNPs could be functionalized with CPPs to improve the transfection of gene materials. For instance, Niu et al. loaded TAT-conjugated AuNPs with miRNA-221-encoded plasmid DNA for melanoma treatment. A skin-penetration test found large amounts of AuNPs in hair follicles, with detectable traces deep into the dermis at ~80 µm. In vivo experiments on tumor-bearing mice showed attenuated tumor growth and apoptosis of tumor cells [98].

Other modalities of inorganic nanoparticles have also been reported for skin delivery. Lio et al. chose mesoporous silica nanoparticles (MSNs) to load siRNA for facile skin cancer treatment [107]. Siu et al. utilized polyetherimide (PEI)-functionalized single-walled carbon nanotubes as siRNA carriers to treat melanoma. They proposed that carbon nanotubes could act as “nano needles” and penetrate cells via a diffusion-like mechanism. In vivo gene silencing as well as tumor inhibition were demonstrated on CD-1 mice [106]. PEI could also be conjugated to lipopolymers such as DSPE-PEG before being non-covalently attached to carbon nanotubes, suggesting a simpler and more expeditious preparation method [133].

#### 3.3.2. Lipid-Based Nanocarriers

Liposomal formulation is among the most-studied lipid-based nanocarriers. Liposomes are generally spherical vesicles containing single or multiple phospholipid bilayers and an aqueous core. Conventional liposomes tend to be rigid and unyielding, rendering it hard for them to penetrate the SC as whole [134]. The encapsulated therapeutics are mostly deposited in superficial skin layers, being released via the exchange of the lipid component between liposomes and the SC. To reach a therapeutic concentration at deeper skin layers, Cevc et al. proposed ultra-deformable lipid vesicles called transfersomes [135]. Transfersomes have been reported to be effective for the transdermal delivery of insulin, inducing hypoglycemia in healthy human volunteers [135]. With polysorbate or sodium cholate as edge activators, transfersomes are expected to squeeze through the paracellular pathways in the SC. In addition to the role of edge activator, sodium cholate could create pores in the SC during its protonation, hence, delivering macromolecular cargo into deeper skin layers [136].

The promising results of transfersomes stimulated the development of novel elastic liposomes. They are endowed with deformability via the incorporation of surfactants to lower the phase-transition temperature of lipid vesicles. These elastic liposomes can be further categorized as niosomes, ethosomes, invasomes, SECosomes, and PEVs. Invasomes are composed of soy phosphatidylcholine, lysophosphatidylcholine, terpenes, and ethanol [137]. As traditional penetration enhancer, ethanol could interact with the polar regions of SC lipids, increasing the lipid fluidity of both the SC and vesicles [138]. Terpenes could also increase lipid fluidity and enhance drug diffusion into the intercellular lipids of SC [137]. Niosomes are self-assembled vesicles, characterized by non-ionic surfactants and cholesterol. Masheswari et al. reported a successful case of using niosomes to encapsulate Hepatitis B surface protein as an antigen and cholera toxin B as an adjuvant for transcutaneous immunization [14]. Even though the particle size of encapsulated niosomes reaches 2.83 ± 0.29 μm, more than half of the loaded antigens were deposited in the deeper SC layers and epidermis, eliciting strong immune response compared to intramuscular inoculation. Ethosomes contain a significant amount of ethanol (20–50%) [139]. Chen et al. designed a cationic ethosomal vesicle for the skin delivery of CPP-conjugated siRNA and achieved 63.2% ± 7.7% target-gene knockdown in mice [115]. The definition and classification of the above elastic liposomal vesicles can slightly vary among different reviews and research articles. Hence, the formulation and vesicle characterization should be given specific attention when cross-evaluating different literature.

#### 3.3.3. Lyotropic Liquid Crystalline Nanodispersions (LLCs)

LLCs are characterized by an ordered internal structure and large interfacial area, serving the purposes of enhanced drug solubility, modulated drug release, and minimized side effects. Lopes et al. firstly reported the reverse hexagonal phase nanodispersion of monoolein (MO) and oleic acid (OA) for the topical delivery of a model peptide, cyclosporin A [124]. Later, several studies introduced PEI into liquid crystals to bind and condense nucleic acids for efficient cutaneous gene delivery. LLC containing MO/OA/PEI formed a disordered hexagonal structure, while the incorporation of siRNA had no effect on the liquid crystalline nanostructure [140]. Depieri et al. further optimized the system by reducing the PEI concentration, and they successfully used it to deliver interleukin-6 siRNA in a psoriasis skin model [125]. Furthermore, Petrilli et al. complexed siRNA with CPPs before adding to MO/OA/PEI LLC. Topical application on a skin inflammation animal model confirmed that the combination of CPPs and nanodispersion brought even higher gene-silencing efficacy than LLCs alone [123].

#### 3.3.4. Dendrimers

Dendrimers embody a central atom core and outwardly branched structure with a large cavity between the branches to incorporate therapeutic molecules. The internal structure and particle size are determined by different preparation methods as well as synthesis generations (G0-10). The surface charge can also be modulated by amine, carboxyl, acetyl, and hydroxyl group modification on the branches [141]. Moreover, CPP conjugation can further enhance permeability. Polyamidoamine (PAMAM) is the most-investigated material for dendrimers. TAT-conjugated PAMAM dendrimers have been reported to load plasmid DNA for transcutaneous vaccination [127,142]. The compact structure of dendrimers ensured protection against DNA degradation. The possible mechanism of skin penetration for dendrimers can be elucidated as follows: (1) dendrimers serve as drug reservoir to boost drug flux into the skin; (2) when dendrimers are composed of penetration-enhancing materials (e.g., IPM), they can perturb the skin via liquefaction or dissolution of SC lipids; and (3) with proper surface modification, dendrimers are prone to accumulating in hair follicles in a time-dependent manner [143].

### 3.4. Microneedles (MNs)

MNs have been emerging as a promising solution for transdermal drug delivery since the 1990s [144]. An MN patch contains an array of micro-scale needles (length < 1000 μm), which can directly pierce through the SC and create transient microchannels for the delivery of various drugs without introducing physical pain [145]. Theoretically, MN platforms cast no limitation over drug molecular weight and hydrophilicity. Therefore, hydrophilic biomacromolecules such as proteins and nucleic acids can be encapsulated into MNs for vaccination as well as the treatment of melanoma, diabetes, and beyond [58,146]. Inorganic materials such as silicon, stainless steel, glass, and ceramics were firstly used to fabricate solid MNs. However, they still pose disadvantages such as insufficient drug payload, a complicated fabrication process, and biohazardous needle sharps. In the past decade, polymers have been highlighted as promising materials for new generations of MNs due to their excellent biocompatibility, versatility, and cost-effectiveness [147]. Polymeric MN insertion ability is governed by the MN fabrication method and MN array geometry (aspect ratio, needle density, base thickness, tip angle, etc.), which are also associated with the pain level perceived by patients [148]. Drug-release rate can also be tuned by adjusting the polymeric material composition.

#### 3.4.1. Dissolvable MNs

Upon insertion, dissolvable MNs are able to quickly dissolve into the skin, which is favorable for instant drug release [149]. Polysaccharides such as dextran, sodium chondroitin sulfate, hydroxypropyl cellulose (HPC), carboxymethyl cellulose (CMC), hydroxypropyl methylcellulose (HPMC), sodium alginate, and hyaluronic acid (HA) contain abundant hydrophilic groups, making them desired substrate polymers for dissolvable MNs [145]. Dissolvable MNs based on chondroitin sulfate and dextran were reported to deliver recombinant human growth hormone in the form of solid dispersion, reaching peak plasma concentration in 15 min after topical application [150]. The addition of lightly cross-linked polymers as well as materials with larger molecular weight could compensate for insufficient mechanical strength. For example, Chen et al. prepared two-layer dissolvable patches from gelatin and sodium CMC for insulin delivery [151]. These patches exhibited sufficient insertion into mouse cadaveric skin under an application force of 9 N, followed by complete dissolution after 1 h [151]. In another case, insulin delivered via starch/gelatin dissolvable MNs were able to attain relative bioavailability of 92% [152]. The shelf-life of insulin in such MNs was reported to be 20–30 days [152,153]. Additionally, the incorporation of low-dose graphene oxide could endow dissolvable MNs with beneficial properties such as enhanced mechanic strength, moisture resistance, and anti-bacterial and anti-inflammatory effects [154].

Dissolvable MNs are compatible to load nanoparticles for more intricate therapeutic purposes. Lopez-Ramirez et al. reported a magnesium (Mg)-microparticle-embedded dissolvable MN patch to actively deliver anti-CTLA-4 antibodies in a melanoma mouse model [155]. In contact with interstitial biofluid, Mg microparticles could instantly generate hydrogen bubbles, which induced significant vortex flow fields at localized sites and served as a “pumping-like” force for deeper and faster dermal delivery [155]. Furthermore, the addition of cowpea mosaic virus nanoparticles into the MNs could vigorously activate the innate immune system and shift the immunosuppressive tumor microenvironment into an immunostimulatory state [156]. As another example, Zeng et al. utilized an MN platform to deliver glucose oxidase (GOx) for tumor starvation therapy. The coated GOx was loaded into dissolvable MNs and demonstrated a catalysis duration of at least 6 days under biological conditions [157].

#### 3.4.2. Biodegradable Polymeric MNs

Biodegradable polymer-based MNs are used to achieve sustained drug release up to months for drugs such as insulin and contraceptives, obviating the inconvenience of frequent administration [158]. Natural biodegradable polymers such as chitosan [159], silk [160], and chitin [161] are considered ideal candidate materials. Wang et al. fabricated insulin-loaded silk fibroin MNs with a sustained insulin release of up to 60 h [160]. However, these patches need to remain adherent to the skin for days to achieve long-period degradation. To address this inconvenience, a combined patch of antigen-loaded degradable MN tips and a dissolvable supporting matrix was reported by Chen et al. [162]. The supporting array was composed of PVP/PVA and quickly dissolved upon insertion, implanting chitosan MN tips in the dermal layer. A sustained release of up to 28 days was achieved in mice [162]. Synthetic degradable polymers including polylactic acid (PLA), polyglycolic acid (PGA), poly(lactic-co-glycolic acid) (PLGA), polycarbonate, polystyrene (PS), and polycaprolactone (PCL) [145] have also been exploited. For further MN modification, one could consider the encapsulation of functional particles such as magnetic graphene quantum dots [163], reduced graphene oxide nanocomposites [164], and graphene quantum dots [165], which render MNs compatible for electroporation or iontophoresis. Biodegradable polymers mostly share the common properties of dissolving in organic solvents or melting at high temperatures. Hence, the fabrication usually involves organic solvents or high temperature to form MNs in micromolds. Sensitive drugs such as peptides, proteins, and nucleic acids are susceptible to such process. Thus, facile fabrication methods such as electro-drawing are to be further developed [166].

#### 3.4.3. Swellable Polymeric MNs

Swellable polymeric MNs are able to absorb large quantities of water in the presence of interstitial fluid and remain insoluble due to the strong chemical or physical chain crosslinking within the polymer matrix. On swelling, they serve as a release-rate-controlling membrane, which can be tuned by modulating the crosslinking density [167]. Polymers that meet the criteria for swellable MN fabrication include poly(hydroxyethylmethacrylate) (PHEMA), acrylate-modified HA (m-HA), polystyrene-block-poly (acrylic acid) (PS-b-PAA), and poly (methyl vinyl ether-co-maleic acid) (PMVE/MA) [168]. Donnelly et al. reported the preparation of PMVE/MA-based MNs, crosslinked with PEG via esterification [169]. PMVE/MA-PEG swelled 3-fold and 50-fold after cross-linking for 24 h and 72 h, respectively. However, cross-linking for the above polymers usually requires high temperature (e.g., 80 °C for PMVE/MA-PEG crosslinking [169]). It is advised that sensitive drugs be post-loaded into the backing adhesive patches as drug reservoirs after the cross-linking process. A mild fabrication method was reported by Yin et al. using 2-ethoxyethanol to endow silk fibroin with swellable and insoluble properties at 25 °C [170]. By increasing the 2-ethoxyethanol/silk fibroin blending ratio, larger swelling capacities up to 800% were shown after insertion, accompanied by porous network formation to facilitate macromolecular drug release [170].

#### 3.4.4. Bio-Responsive MNs

**Figure 4:**
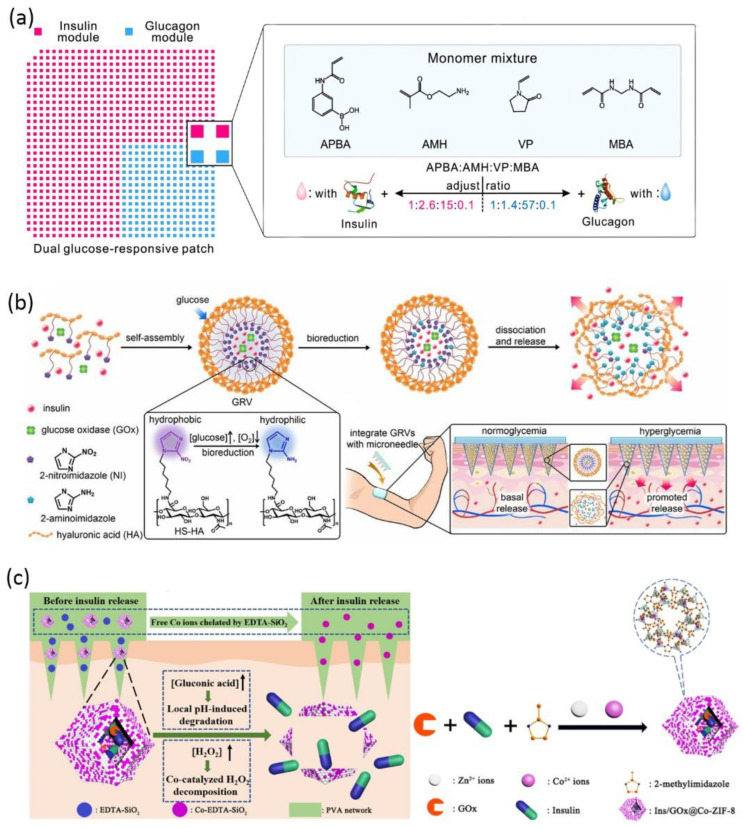
Schematic of glucose-responsive insulin delivery microneedles: (**a**) A dual-module microneedle patch to separately encapsulate insulin and glucagon. The two modules comprise different ratios of key monomers, synergistically responding to both hyperglycemic and hypoglycemic states (reproduced with permission from Wang et al., Proceedings of the National Academy of Sciences of the United States of America; published by National Academy of Sciences, 2020) [174]; (**b**) fast-responsive MN patch loaded with hypoxia-sensitive hyaluronic acid (HS-HA) vesicles containing insulin and GOx. In localized hypoxic environment, the hydrophobic 2-nitroimidazole groups of HS-HA are reduced to hydrophilic 2-aminoimdazole, leading to the dissociation of vesicles and subsequent release of insulin (reproduced with permission from Yu et al., Proceedings of the National Academy of Sciences of the United States of America; published by National Academy of Sciences, 2015) [179]; (**c**) the insulin and glucose oxidase are encapsulated in metal–organic framework and further loaded into the microneedle matrix. The low pH induced by glucose oxidation leads to framework degradation and insulin release. The preloaded Co^2+^ catalyzes the decomposition of the byproduct H_2_O_2_. The excessive Co^2+^ is chelated by EDTA-SiO_2_ nanoparticles in the microneedle matrix (reproduced with permission from Yang et al., ACS Applied Materials & Interfaces; published by ACS, 2020) [176].

The unique physiological disease microenvironment is utilized to trigger drug release from bio-responsive MNs, including pH, glucose, hypoxia, temperature, enzymes, and receptors [147]. Glucose-responsive insulin delivery is the most-discussed solution for regulating blood glucose without inducing hypoglycemia [171]. Bio-responsive MN is a versatile platform to mimic pancreatic endocrine function either by incorporating glucose-sensitive matrix material or by encapsulating stimuli-responsive nanoparticles to adjust insulin release according to endogenous glucose level. For the former strategy, cationic polymers modified with phenylboronic acid were reported to release insulin via a glucose-triggered charge switch [172,173]. MNs were prepared via in situ photopolymerization of the insulin and matrix monomer premixture, leading to 100% insulin encapsulation and a high loading capability. The consecutive application of such coin-like MN patches successfully maintained plasma glucose level within the normal range for 48 h in both diabetic mice and minipigs [172]. Wang et al. further designed a dual-module MN patch to separately encapsulate insulin and glucagon, mimicking the counterregulatory effects of β and α cells in the pancreas (Figure 4a) [174]. The two modules were featured by different ratios of key monomers, hence, allowing a synergistic response in both hyperglycemia and hypoglycemia conditions [174]. For the second strategy, glucose oxidase (GOx) is frequently incorporated into nanoparticles as glucose sensors to modulate insulin release after microneedle application. GOx is able to catalyze glucose into gluconic acid with H_2_O_2_ as byproduct. Hence, the glucose-induced acidic or hypoxic microenvironment [175,176] and H_2_O_2_ [171,177,178] can be considered as stimuli to trigger insulin release. For example, hypoxia-sensitive hyaluronic acid vesicles containing insulin and GOx were incorporated into MN to achieve fast responsiveness to high glucose level (Figure 4b) [179]. Further research work suggested preloading Co^2+^ in MNs to catalyze the decomposition of the harmful byproduct H_2_O_2_ (Figure 4c) [175].

## 4. Outlook

The skin delivery of biomacromolecules has promising clinical relevance for both localized and systemic treatment. However, booming pre-clinical research contrasts with the sporadic clinical investigations. There stand a series of challenges to tackle for successful lab-to-market translation. Considering the labile nature of biological macromolecules, a detailed investigation regarding their stability during formulation preparation, as well as different periods of storage, is necessary. Next, a dose regimen with high efficacy and low irritation should be screened. To this end, both drug-release behavior and pharmacokinetics should be systemically characterized. Unveiling the currently obscured mechanisms of different enhancers also contributes to more rational formulation design. In addition, extensive research should include the cumulative side effects associated with penetration enhancers and drug carrier materials. Eventually, scale-up production with low cost and prolonged shelf-life of biologics at room temperature are the key to gaining advantages in the pharmaceutical landscape.

## Figures and Tables

**Table 1:** Current reports of using nanovesicles for dermal and transdermal delivery of biomacromolecules.

Nanocarrier	Composition	Particle Size/ζ-Potential	Cargo	Indication	Ref.
Gold nanoparticle	AuNPs in Pluronic F-127 gel	190~208 nm/+30~ +45 mV	PGT DsiRNA	Diabetic wound-healing	[97]
	AuNPs with PEI and TAT conjugation	199 ± 7.76 nm/16.81 ± 0.56 mV	pDNAs encoding microRNA-221 inhibitor gene	Melanoma	[98]
	AuNPs modified with thiolated siRNA and PEG coating	12.38 ± 1.59 nm/~28 mV before coating	EGFR siRNA	Psoriasis	[99]
	AuNPs modified with thiolated siRNA and thiolated oligoethylene glycol	28 ± 3 nm/-	GM3S siRNA	Diabetic wound-healing	[100]
	AuNPs with PEI and LL37 coating	7.6 ± 0.9 nm/36.8 ± 2.3mV	pDNA encoding VEGF	Diabetic wound-healing	[101]
	AuNP-CONH-VEGF	11.2nm ± 0.1 nm/-	VEGF	Wound healing	[102]
	AuNP-CONH-KGF	65.7 nm/− 34.9 mV	KGF	Wound healing	[103]
	AuNPs	11.6 nm/18.3 mV	HRP, β-gal, OVA	-	[104]
	AuNP-PEG- Esc(1-21)	~14 nm/−35.58 mV	Antimicrobial peptide Esc(1-21)	Antipseudomonal wound healing	[105]
Carbon nanotube	PEI functionalized carbon nanotubes	-/40~60 mV	BRAF siRNA	Melanoma	[106]
Mesoporous silica nanoparticle	Mesoporous silica nanoparticles with poly-L-lysine coating	200 nm/−34 mV	TGFβR-1 siRNA	Facile skin cancer	[107]
Nanostructured lipid carrier	0.5% glycerol distearate, 0.25% oleic acid, 0.25% PEI, 1.0% Poloxamer 407, and pH7.4 phosphate buffer	230 nm/+10 mV	TNFα siRNA	Psoriasis	[108]
	DOTAP/sodium cholate/coiled-coil protein = 60:10:7	174.22 ± 8.71 nm/34.5 ± 1.7 mV	Keap1 siRNA	Diabetic wound-healing	[109]
	Elastic liposomes. DOTAP/DOPE/Cholesterol = 6:4.2:1.8(w/w/w)	147.7 ± 31.9 nm/46.7 ± 13.4 mV	antagomiR-203 or SOCS3 siRNA	Psoriasis	[110]
	Elastic liposomes. Soya phosphatidylcholine/span 80 = 86:14 (w/w)	122 ± 9.2 nm/-	P. falciparum surface antigen, MSP-1_19_	Malaria vaccine	[17]
	Elastic liposomes. Soya phosphatidylcholine/span 80 = 86:14 (w/w)	123.8 ± 51.31 nm/9.36 mV	Recombinant fusion protein PfMSP-Fu_24_	Malaria vaccine	[111]
	Elastic liposomes. HPC/cholesterol/DOTAP = 8:4:1 (molar ratio)	107 ± 0.757nm/56.5 ± 1.13mV	Growth factors fused with low-molecular-weight protamine	Diabetic wound-healing	[5]
	SECosomes. DOTAP/DOPE/NaChol = 6:1:1(w/w/w), rehydrated in 30% ethanol	172 nm/44 mV	DEFB4 siRNA	Psoriasis	[112]
	Deformable cationic liposomes. Octadecylamine/cholesterol = 10:1	208.5 ± 11.5 nm/-	pDNA encoding HBsAg	Hepatitis B vaccination	[113]
	Niosomes. Span85/cholesterol = 7:3	2.3 ± 0.15 μm/-	pDNA encoding HBsAg	Hepatitis B vaccination	[114]
	Ethosomes. DOTAP/cholesterol = 5:1, with SPACE modification	108.4 ± 3.4nm/49.1 ± 0.6mV	GAPDH-siRNA-SPACE conjugate	-	[115]
	Pyrrolidinium lipid/1,2-di-(9Z-octadecenoyl)-snglycero-3-phosphocholine/DOPE/DSPE-PEG2000 = 1:2:2:0.2	102 ± 6 nm/32.14 ± 6.21 mV	STAT3 siRNA and TNFα siRNA	Psoriasis	[116]
	Lipidoid 306O_13_/DSPC/cholesterol/C14-PEG = 50:10:38.5:1.5ao	110 nm/-	TNFα siRNA	Diabetic wound-healing	[117]
	DOPC and cholesterol-conjugated oligonucleotides	21 ± 2 nm	IL17RA gapmer antisense oligonucleotide	Psoriasis	[11]
	Poloxamer 188/Tween 80/Precirol^®^ ATO 5/Miglyol^®^ 812 N (1:2:10:1)	273.6 ± 27.64 nm/~31.63 ± 1.9 mV	Antimicrobial peptide LL37	Wound healing	[118]
	Lipidic blend containing Precirol^®^ ATO 5/Miglyol^®^ 182 (10:1), emulsified with 0.67% (w/v) Poloxamer and 1.33% (w/v) polysorbate 80	335 nm/-27 mV	Recombinant human epidermal growth factor	Wound healing	[119]
Hybrid lipid–polymer nanoparticle	Inner PLGA core coated with cyclic head lipid/DOPC/DSPE-PEG2000(4.0/4.5/1.5, molar ratio)	163 ± 9 nm/35.14 ± 8.23 mV	TNFα siRNA	Skin inflammation	[120]
	2.0% of Compritol^®^ 888 ATO (lipid), 1.5% of poloxamer 188 and 0.1% of the cationic polymer poly(allylamine hydrochloride)	142 nm/+25 mV	TNFα siRNA	Psoriasis	[121]
Liquid crystalline nanodispersion	MO:OA:PEI:Aqueous phase(Tris-HCl) = 8:2:1:89 or 8:1:0.5:90.5 (w/w/w/w)	220 nm/1 mV or 170 nm/− 2 mV	TyRP-1 siRNA	Vitiligo	[122]
	MO/OA/PEI/aqueous phase 8/2/1/89 w/w/w/w, functionalized with TAT	310 ± 8 nm/1.19 ± 0.27 mV	TNFα siRNA	Inflammation	[123]
	MO/OA/poloxamer/aqueous phase 8:2:0.9:89.1 w/w/w/w	181.77 ± 1.08 nm/-	Cyclosporin A	-	[124]
	MO/OA/PEI/aqueous phase 8/2/1/89 w/w/w/w	215.4 ± 7.9 nm/0.7 ± 1.0 mV	IL-6 siRNA	Psoriasis	[125]
	Poloxamer 407 containing 0.5% liquid crystalline gel (glycerol monooleate/water 70:30)	~130 nm/+3~ +11 mV	Antimicrobial peptide LL37	Wound healing	[126]
Dendrimer	TAT-conjugated PAMAM	106 nm/+45 mV	pDNA encoding GFP	DNA vaccine	[127]
Non-ionic colloidal carrier system	DMSO/IPM/Tween 80/Span 20 (0.45/2.5/0.3/0.2, v/v/v/v)	100.6 ± 28.3 nm/-	Insulin	Diabetes	[128]
SAMiRNA (self-assembled micelle)	PEG and hydrocarbon conjugation at each end of unmodified oligonucleotides	<100 nm/neutral	Dual-conjugated DNA/RNA heteroduplex	Androgenetic alopecia	[129]
Polysaccharide nanoparticles	Panax quinquefolium polysaccharide	20 nm/-	Panax quinquefolium polysaccharide	UVB-induced skin cancer	[130]
Ginsenoside nanoparticles	Ginsenosides/insulin (25:1, w/w) self-assembly	165.5 ± 0.6 nm/-	Insulin	Diabetes	[131]

## Data Availability

Data sharing not applicable.
